# Comparative Analysis of the Transcriptome of the Chicken Breast Muscle at Different Developmental Stages

**DOI:** 10.3390/ani16071071

**Published:** 2026-04-01

**Authors:** Yuting Jin, Xiaodong Tan, Lu Liu, Jiahua Li, Jie Dong, Minjie Huang, Ayong Zhao, Deqian Wang

**Affiliations:** 1 College of Animal Science and Technology & College of Veterinary Medicine, Zhejiang Agriculture and Forestry University, Hangzhou 311300, China; 2 Institute of Animal Husbandry and Veterinary Science, Zhejiang Academy of Agricultural Sciences, Hangzhou 310021, China; 3 Key Laboratory of Livestock and Poultry Resources (Poultry) Evaluation and Utilization, Ministry of Agriculture and Rural Affairs, Hangzhou 310021, China

**Keywords:** chicken, breast muscle, RNA-seq, development

## Abstract

Chickens are a primary source of protein in the human diet, with demand increasing annually. However, research on genes that promote chicken meat development remains relatively limited. Therefore, in this study, breast muscle samples (pectoralis major, *n* = 10 per group) from chickens at five developmental stages (D1, D35, D70, D105, and D140) were selected to investigate genetic-level changes and identify additional genes influencing chicken meat development. Differential expression analysis between adjacent stages (|FC| > 1.5, *p* < 0.05) revealed 42 differentially expressed genes (DEGs) shared across four comparisons, primarily enriched in muscle development pathways such as focal adhesion, the regulation of the actin cytoskeleton, and Wnt signaling. Weighted gene coexpression network analysis (WGCNA) revealed that four modules were significantly correlated with body weight and breast muscle weight phenotypes. By integrating the hub genes of the four modules and the DEGs, we identified key genes, including *MEGF10*, *MYOM2*, *TM4SF1*, *HNMT*, *NR4A3*, and *Wnt5a*. Furthermore, we conducted a comparative analysis of key gene expression trends across commercial broilers (CBs) and Beijing You (BJY) chickens. *MYOM2* and *Wnt5a* exhibited distinct expression patterns during the early developmental stage of Xianju (XJ) chickens, suggesting that these genes may be critical factors distinguishing XJ chicken breast muscle development from that of other breeds. In all three chicken breeds analyzed, the expression levels of *HNMT* and *MEGF10* gradually decreased with increasing age, indicating that their functions are universal in poultry muscle development. In summary, our findings revealed key regulatory genes that influence breast muscle development, offering candidate targets for marker-assisted selection in poultry breeding programs.

## Simple Summary

Breast muscle samples were collected from Xianju chickens at five developmental stages for RNA-Seq analysis to identify candidate genes influencing breast muscle development. Comparing gene expression between adjacent growth stages yielded 4939, 1594, 586, and 3072 differentially expressed genes (DEGs), involving multiple pathways related to muscle development, including focal adhesion and the regulation of the actin cytoskeleton. Weighted gene coexpression network analysis revealed that four modules were significantly correlated with body weight and breast muscle weight phenotypes. By integrating the hub genes of the four modules and the DEGs, we identified key genes, including *MEGF10*, *MYOM2*, *TM4SF1*, *HNMT*, *NR4A3*, and *Wnt5a*. Furthermore, *HNMT* and *MEGF10* exhibited expression patterns in the breast muscle tissues of commercial broilers and Beijing You chickens that were consistent with those observed in Xianju chickens.

## 1. Introduction

Chicken meat is a high-quality, economical animal protein source due to its high-protein, low-fat content and low production costs [1]. Skeletal muscle accounts for approximately 40% of an animal’s total body weight and is a key factor influencing poultry meat production performance [2]. Additionally, chickens have a short growth cycle and exhibit distinct muscle development characteristics, making them an ideal model for myogenesis research [3]. Skeletal muscle development is influenced by multiple factors. For example, compared with 100% roasted SBM, the inclusion of 100% peanut meal in poultry feed significantly increases body weight and carcass weight in chickens [4]. In addition to feeding management, genetic factors also play an important role in regulating chicken muscle growth and development. For instance, a comparative study between Jingxing Yellow (JX) chickens selectively bred for high breast muscle weight and the original JX chickens revealed that *IGF2BP1* mRNA levels were negatively correlated with breast muscle weight, suggesting that this gene may function as an important negative regulator [5]. *MUSTN1* is associated with chicken embryonic development and skeletal muscle growth, and it has been shown to promote the proliferation and differentiation of chicken skeletal muscle satellite cells [6].

The growth and development of poultry skeletal muscle can be divided into two stages: the formation of muscle fibers during the embryonic period and the development of muscle fiber hypertrophy after birth. During embryonic development, the paraxial mesoderm generates somite plates, which subsequently form somites [7]. Among these, dorsal somites differentiate into mononuclear myoblasts, which ultimately differentiate into muscle fibers through processes such as proliferation, migration [8,9]. In chickens, somite formation is initiated at embryonic day 2.5, with the population of muscle fibers reaching a plateau by embryonic day 20, indicating that embryonic myogenesis is largely complete [10,11]. Posthatch skeletal muscle development in poultry primarily involves myofibrillar hypertrophy mediated by protein accumulation and satellite cell fusion [12]. The above-mentioned process of tissue development is regulated by multiple factors, involving the activation of signaling pathways, the action of transcription factors, and changes in downstream gene expression [13]. *IGF-1* can increase skeletal muscle protein synthesis through the PI3K/Akt/mTOR pathway [12,14]. Research has demonstrated that infusing IGF-1 into fasted chickens increases the rate of breast muscle protein synthesis [15]. Furthermore, seven days after injecting a retrovirus encoding IGF-I into the hindlimbs of chicken embryos, the gastrocnemius muscle area was 80% larger than that of the corresponding region in the control group [16]. Stained sections revealed that overexpression of IGF-1 increased the total number of muscle fibers [16]. The overexpression of multiple genes can promote muscle hypertrophy. For instance, FilaminC mRNA expression is higher in chicken myotubes than in chicken myoblasts, and its overexpression enhances muscle hypertrophy [17]. However, current research on related genes focuses primarily on comparisons of differences at a single time point, with limited exploration of the functional mechanisms of these genes during the dynamic process of poultry muscle development.

Gene expression levels fluctuate across different developmental stages in animals. Li et al. [18] demonstrated that distinct hub genes are involved in muscle growth and development across various growth stages in Jinmao Hua chickens. At 6 weeks of age, the *FGF16* gene was identified as the sole hub gene closely associated with muscle growth, whereas at 10 weeks, *FGF10*, *SOCS2*, *CTSG*, and others were selected as hub genes [18]. RNA-seq analysis of skeletal muscle development across three prenatal stages and one neonatal stage in goats revealed that DEGs enriched in biological pathways such as the Rap1 signaling pathway and PI3K-Akt signaling pathways were directly correlated with temporal changes in skeletal muscle development [19]. Cao et al. [20] performed RNA-seq on muscle samples from Pekin duck and Hanzhong Ma duck embryos at 17, 21, and 27 days post-hatching, as well as at 6 months post-natally. They reported that DEGs, including *ARRDC2*, were coexpressed across all developmental time points, and the focal adhesion and ECM-receiver interaction pathways were the most enriched across all developmental stages, involving DEGs such as *MYL9* and *PAK1* [20]. DNA methylation plays a crucial role in muscle development. Ran et al. [21] reported a significant increase in the number of differentially methylated regions at embryonic day 17 (E17), suggesting that E17 may represent a critical stage for skeletal muscle development in chicken embryos. Concurrently, differentially methylated genes at E11 and E17 (e.g., *MYOG*, *CFL2*, and *MEF2D*) were enriched in pathways closely associated with muscle development, suggesting that they may play important roles in embryonic muscle development in chickens [21].

The Xianju (XJ) chicken is a high-quality native Chinese breed, serving as a dual-purpose breed for both meat and egg production. It is renowned for its tender meat, rich flavor compounds, and strong adaptability, reaching market weight at 140 days of age [22]. The average market price of XJ chickens is approximately 68 RMB per kilogram, while that of ordinary chickens is only 38 RMB per kilogram. Therefore, XJ chickens have high economic value. XJ chickens have an average feed conversion ratio (FCR) of 3.2–3.31. Compared with Wannan Yellow chickens, the FCR of XJ chickens is 0.47–0.82 lower [23]. At 140 days of age, their body weight is approximately 1510 g, carcass weight is approximately 1280 g, and breast muscle weight is approximately 180 g. Due to their higher intramuscular fat content and better meat quality than males [24], female XJ chickens were chosen for this study. Transcriptome sequencing was performed on breast muscle samples collected at five developmental stages. This study aims to identify key genes regulating breast muscle development in XJ chickens through differential expression analysis and weighted gene co-expression network analysis (WGCNA). We further investigate the expression dynamics of these key genes during breast muscle development in XJ chickens. Additionally, we conducted comparative analyses of key gene expression patterns across commercial broilers (CBs) and Beijing You (BJY) chickens to explore both conserved and breed-specific regulatory mechanisms of muscle development under different genetic backgrounds. We hypothesize that the molecular regulatory mechanisms underlying breast muscle development in XJ chickens share conserved pathways with those in CB and BJY while also exhibiting breed-specific regulatory characteristics. Through systematic elucidation of these questions, this study aims to provide a theoretical basis and candidate gene resources for molecular marker-assisted breeding of XJ chickens.

## 2. Materials and Methods

### 2.1. Animals and Sample Collection

The XJ chickens (female) used in this study were sourced from Sishan Farm (Hangzhou, China). During the entire rearing period, starter feed is provided from 1 to 56 days of age, and grower feed is provided from 57 to 140 days of age. Both feeds are in pellet form. Fresh feed and water were available *ad libitum*. From 1 to 7 days of age, Xianju chickens were reared under an environmental temperature of 33 °C, which was then gradually reduced to room temperature. The relative humidity is between 55% and 60%. Light intensity was set between 10 and 12 lux, with a lighting duration of 20–24 h, which was gradually decreased to natural light. The experimental chickens were housed in a windowed, enclosed building. From 1 to 90 days of age, they were reared in group cages with staged separation, and from 90 to 140 days of age, they were housed individually in single cages. At 1, 35, 70, 105, and 140 days of age (D1, D35, D70, D105, and D140), 20 randomly selected Xianju chickens were fasted for 12 h before they were weighed and slaughtered. The test chickens were euthanized using the CO_2_ inhalation method [25]. Post-slaughter measurements included measurements of breast muscle weight (BMW) and other traits. The collected breast muscle tissue was rapidly frozen in liquid nitrogen and stored at −80 °C. Ten samples per group were randomly selected for transcriptomic sequencing.

### 2.2. Total RNA Isolation, Library Preparation, and mRNA Sequencing

Total RNA was extracted using TRIzol kits (Invitrogen, Carlsbad, CA, USA). RNA concentration and integrity were determined using Nanodrop^®^ series spectrophotometers (Thermo Fisher Scientific, Waltham, MA, USA) and the Agilent^®^ 2100 Bioanalyzer (Agilent Technologies, Inc., Santa Clara, CA, USA), respectively, and verified by agarose gel electrophoresis. A Ribo-Zero™ Gold Kit (Illumina, Inc., San Diego, CA, USA) was used to remove rRNA while preserving mRNA. Enriched mRNA was randomly fragmented and reverse-transcribed into cDNA. The cDNA was subsequently subjected to purification, end repair, poly(A) tailing, and adapter ligation. The final cDNA library was subsequently sequenced using the Illumina NovaSeq 6000 platform (Illumina, Inc.).

### 2.3. Quality Control and Genome Mapping

The quality of the raw data was checked using FastQC (v0.11.9), and low-quality reads were removed using Fastp (v0.23.4) (-q 20 -u 30 -n 5 -l 150) to obtain clean reads [26,27]. HISAT2 v2.2.1 (-rna-strandness RF) [28] was subsequently used to compare the qualified reads to the chicken reference genome GRCg7b (GCF_016699485.2). File conversion, sorting, indexing, and quality control were performed using SAMtools (v1.21) [29]. The filtered reads were assembled using StringTie (v2.2.3) [30]. The raw gene count matrix was obtained according to the Python (v3.12) script provided by StringTie (l = 150) and normalized via the TPM method.

### 2.4. Analysis of Differentially Expressed Genes and Expression Trends

Protein-coding gene expression levels were normalized by DESeq2 (v4.2.2) [31], and differentially expressed genes (DEGs) were identified between adjacent time points on the basis of raw transcript counts (|FC| > 1.5, *p* < 0.05). Trend analysis of the DEGs was performed using short time series expression miner software, and the results were visualized via the OmicShare tool (https://www.omicshare.com/, accessed on 4 January 2026).

### 2.5. Analysis of the Weighted Gene Coexpression Network

To identify genes associated with breast muscle development, WGCNA was employed to construct a coexpression network based on mRNA transcriptional abundance in breast muscle tissue. A soft threshold (β = 14) was determined for mRNA on the basis of the scale-free distribution results (R^2^ > 0.85). The coexpression network was subsequently constructed, and gene modules were identified using the stepwise dynamic pruning method, with parameters set as follows: minModuleSize = 50 and mergeCutHeight = 0.25. A correlation coefficient > 0.5 was set as the screening threshold for important gene modules in the breast muscle. Within these modules, DEGs with a |KME| > 0.8 were defined as hub genes.

### 2.6. Functional Enrichment Analysis

To investigate the potential biological functions and associated pathways of the candidate DEGs, we conducted analyses on the basis of the Kyoto Encyclopedia of Genes and Genomes (KEGG) database and visualized the results using the online platform OmicShare. Pathways with *p* < 0.05 were considered significantly enriched.

### 2.7. Comparison of Gene Expression Between Commercial Broilers and Local Chickens

To validate the cross-breed universality of key gene expression patterns, we selected breast muscle samples from commercial broilers (CBs, *n* = 6 at each time point) and Beijing You (BJY, *n* = 6 at each time point) chickens at days 1, 43 and 91, along with muscle samples from XJ chickens at D140 with high-breast-muscle-weight (HBM, *n* = 10) and low-breast-muscle-weight (LBM, *n* = 10) groups. On the basis of the above pipeline, we obtained gene expression data from these populations. DESeq2 software (v4.2.2) [31] was used for differential expression analysis of protein-coding genes (|FC| > 1.5, *p* < 0.05).

### 2.8. Validation of Hub Genes

To verify the sequencing results, we performed real-time PCR (RT-PCR) to determine the relative expression of the selected genes (*HNMT*, *NR4A3*, *MYOM2*, *TM4SF1*, and *Wnt5a*). Total RNA was extracted using TRIzol reagent (Invitrogen), and cDNA was obtained by reverse transcribing the total RNA with a FastQuant RT Kit (Tiangen, Beijing, China). Each sample was replicated three times. All primers were designed using Oligo 6.0 software; the primer details are provided in Supplementary Table S1. The data were analyzed using the 2^−ΔΔCT^ method, with *RPL32* and *HSPA2* serving as reference genes.

### 2.9. Statistical Analysis

The Shapiro–Wilk test was used to test for normality in each group, and *p* > 0.05 indicated a normal distribution. Normally distributed data were analyzed using the independent samples *t*-test, whereas non-normally distributed data were analyzed using the Mann–Whitney *U* test. Pearson correlation analysis was employed to examine the correlation between gene expression levels and phenotypes. All the statistical analyses were performed using SPSS 25.0 software. *p* < 0.05 indicated significant differences.

## 3. Results

### 3.1. Analysis of Phenotypic and Sequencing Data

**Figure 1:**
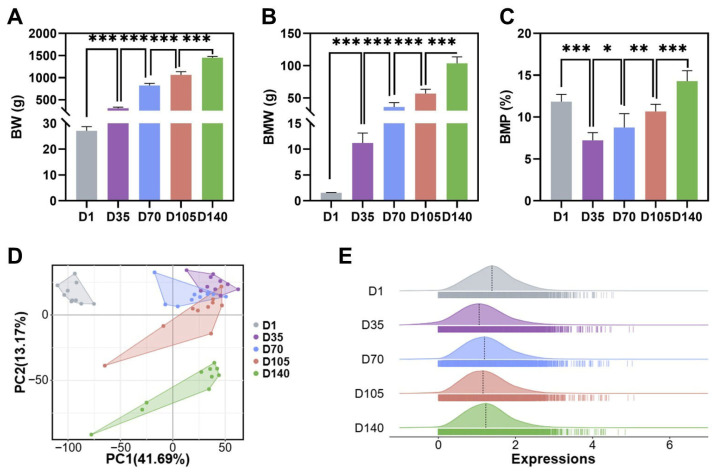
Profiles of breast muscle phenotypes and sequencing results across different developmental stages: (**A**) Comparative statistics of body weight (BW) at different developmental stages. * indicated *p* < 0.05, ** indicated *p* < 0.01, *** indicated *p* < 0.001. (**B**) Comparative statistics of breast muscle weight (BMW) at different developmental stages. (**C**) Comparative statistics of breast muscle percentage (BMP) at different developmental stages. (**D**) Distribution of breast muscle samples across the five developmental stages. (**E**) Distribution of gene expression levels across five developmental stages. The *x*-axis means the TPM values of genes.

Phenotypic results revealed significant differences in body weight (BW), BMW, and breast muscle percentage (BMP) across developmental stages, with BMP reaching its maximum at D140 (Figure 1A–C, Table S2). The average body weight gain was highest between D35 and D70, with an average daily gain of 14.88 g. The highest average breast muscle weight gain occurred between D105 and D140, with an average daily gain of 1.64 g (Table S3). The growth rate continuously decreased from 5.14% to 1.28%. After quality control was completed, 50 breast muscle samples were subjected to transcriptome sequencing, yielding 230 Gb of clean data after quality control, and an average reference genome mapping rate of 92%. After filtering, the average Q30 reached 96.56%. Genes with an average tpm > 1 were selected, resulting in a total of 10,579 genes. PCA analysis revealed that samples from different time points clustered together, with samples from D1 and D140 completely separated from those from the other three time points (Figure 1D,E).

### 3.2. Analysis of DEGs at Different Stages

**Figure 2:**
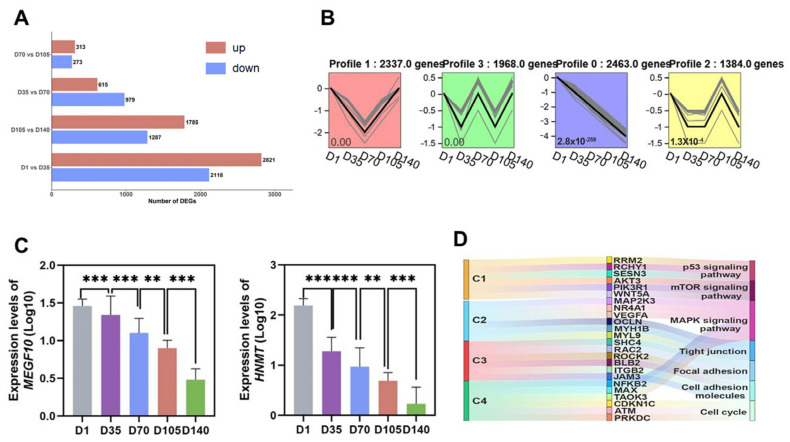
Differential expression analysis and functional prediction of genes: (**A**) Statistics of differentially expressed genes (DEGs) across five stages. (**B**) Analysis of gene expression trends. (**C**) *MEGF10* and *HNMT* expression levels across five developmental stages. ** indicated *p* < 0.01, *** indicated *p* < 0.001. (**D**) Functional enrichment pathways of DEGs across different comparison groups. C1: D1 vs. D35, C2: D35 vs. D70, C3: D70 vs. D105, C4: D105 vs. D140.

Differential expression analysis of genes between adjacent time points yielded 4939 (D1 vs. D35, C1), 1594 (D35 vs. D70, C2), 586 (D70 vs. D105, C3), and 3072 (D105 vs. D140, C4) DEGs (Figure 2A, Table S4). Trend analysis of all genes revealed significant temporal expression changes in profiles 0, 1, 2, and 3, with genes in profile 0 showing downregulation with increasing age (Figure 2B). Among the four comparison groups, 42 identical DEGs were identified, with 14 genes, including *MEGF10*, *TM4SF1*, and *HNMT*, exhibiting similar expression trends (Figure 2C). We performed KEGG enrichment analysis on the DEGs and found that pathways such as the p53 and mTOR signaling pathways were enriched in C1 and C2, whereas pathways including cell adhesion molecules and tight junctions were enriched in C3 and C4 (Figure 2D, Table S5). All four comparison groups were enriched in eight pathways, including focal adhesion, apoptosis, and the regulation of the actin cytoskeleton.

### 3.3. Identification of Hub Genes via WGCNA

**Figure 3:**
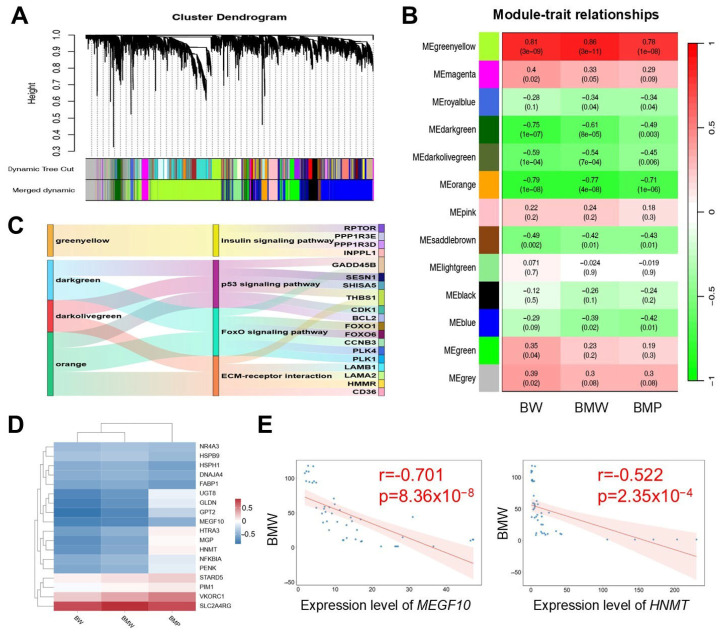
Weighted gene coexpression network analysis (WGCNA) and the identification of hub genes: (**A**) Clustering and merging results for gene modules in the WGCNA pipeline. (**B**) The relationship between BW/BMW/BMP and gene modules. (**C**) Functional enrichment pathways of candidate genes across different modules. (**D**) Correlation analysis between hub genes and phenotypic traits. (**E**) Correlation analysis between *MEGF10*/*HNMT* expression and BMW.

WGCNA was employed to identify genes associated with the breast muscle phenotype. The results indicated that genes exhibiting similar expression patterns in breast muscles were clustered into 13 modules. Among these modules, the greenyellow module was significantly positively correlated with BW and BMW, whereas the darkgreen, darkolivegreen, and orange modules were significantly negatively correlated with these phenotypes (Figure 3A,B). The four modules contained 3068, 290, 101, and 255 genes, respectively. Genes with a |KME| > 0.8 were subsequently selected as candidate genes for KEGG analysis (Table S6). The results revealed significant pathway enrichment in the greenyellow, darkgreen, darkolivegreen, and orange modules, with 21, 14, 15, and 17 pathways enriched, respectively (Figure 3C). Specifically, the p53 signaling pathway was enriched in genes from the darkgreen, darkolivegreen, and orange modules, and the FoxO signaling pathway was enriched in genes from the darkgreen and orange modules. In the greenyellow module, genes such as *INPPL1*, *PRKAG1*, and *PRKAG3* participated in the insulin signaling pathway (Table S7). Finally, screening for DEGs with a |KME| > 0.8 yielded 18 hub genes, among which genes such as *HNMT*, *NR4A3*, and *MEGF10* were significantly correlated with BW and BMW (Figure 3D,E, Table S8).

### 3.4. Expression Trends of Key Genes in Different Chicken Breeds

**Figure 4:**
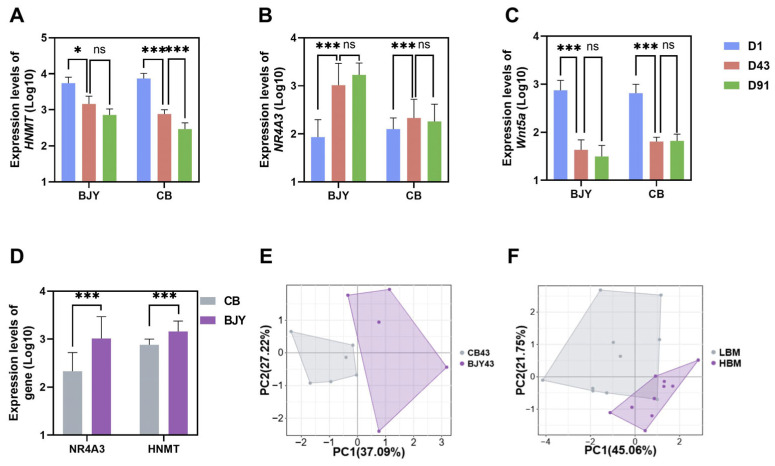
Gene expression analysis of breast and leg muscles in commercial broilers (CBs) and Beijing You (BJY) chickens at different developmental stages, and gene expression analysis in breast muscles of 140-day-old Xianju (XJ) chickens: (**A**–**C**) Differential expression analysis of *HNMT*, *NR4A3*, and *Wnt5a* genes across different days of age in CB and BJY chickens. (**D**) Differential expression analysis of *NR4A3* and *HNMT* genes in breast muscle samples between CB and BJY chickens at 43 days of age. ns indicated *p* > 0.05, * indicated *p* < 0.05, *** indicated *p* < 0.001. (**E**) Distribution of samples for breast muscle in CB and BJY chickens at 43 days of age. (**F**) Distribution of samples for high and low breast muscle weight in XJ chickens at 140 days of age.

Differential analysis of the breast muscle samples revealed that *HNMT* expression significantly decreased with increasing age in BJY, CB, and XJ, whereas *MEGF10* expression significantly decreased with increasing age in BJY and XJ. *NR4A3* expression was upregulated during early development in CB and XJ. *TM4SF1* expression increased in BJY from D43 to D91, with a similar upward trend observed in XJ during the same developmental period. Additionally, during early development, *MYOM2* expression was upregulated in BJY and CB but downregulated in XJ. Conversely, *Wnt5a* expression decreased in BJY and CB but tended to increase in XJ (Figure 4A–C). When the two breeds were compared at D43, compared with CB, BJY presented higher *HNMT* and *NR4A3* expression, with no significant differences observed for the expression of other genes (Figure 4D,E, Figures S1 and S2). In the comparison of the HBM and LBM at D140, *MYOM2* and *TM4SF1* were significantly higher in the HBM than in the LBM, whereas *NR4A3* and *HNMT* showed the opposite pattern (Figure 4F and Figure S3).

In leg muscle samples, *NR4A3* expression was upregulated in BJY and CB during early development, whereas *Wnt5a* expression showed the opposite pattern. From D43 to D91, only CB showed significant upregulation of the expression of *MEGF10*. Furthermore, in comparisons between the two breeds at the same age, only *NR4A3* expression at D43 was significantly higher in CB than in BJY, with no other significant differences observed.

### 3.5. Validation of the Hub Genes via RT-PCR

**Figure 5:**
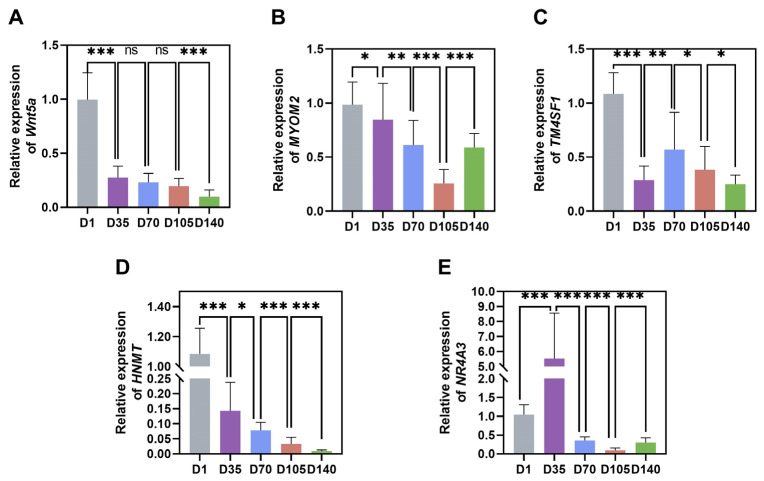
Validation of mRNA sequencing by RT-PCR: (**A**–**E**) Expression of *NR4A3*, *MYOM2*, *TM4SF1*, *HNMT*, and *Wnt5a* at different developmental stages. ns indicated *p* > 0.05, * indicated *p* < 0.05, ** indicated *p* < 0.01, *** indicated *p* < 0.001.

To confirm the expression levels of the sequenced RNA molecules, we performed RT-PCR on the hub genes (*NR4A3*, *MYOM2*, *TM4SF1*, *HNMT*, and *Wnt5a*). The results revealed that *HNMT* expression levels significantly decreased with increasing age, whereas *MYOM2* expression gradually decreased between D1 and D105 before it rebounded. The expression of *TM4SF1* and *Wnt5a* peaked at D1. *NR4A3* expression peaked at D35, subsequently decreased, and slightly increased at D140. Overall, the RT-PCR results were consistent with the sequencing data (Figure 5).

## 4. Discussion

Poultry meat production is projected to account for 47% of total meat production by 2030 [32]. Chicken production can be influenced by multiple factors. In addition to nutritional regulation through additives such as guanidinoacetic acid, *Castanea sativa* tannin, and phosphatidylethanolamine, genetic background is also an important aspect that cannot be overlooked [33,34,35]. After hatching, muscle growth in poultry primarily depends on muscle fiber hypertrophy, a process regulated by multiple genes. This study employed transcriptome sequencing of chicken breast muscles at 1, 35, 70, 105, and 140 days of age to explore key signaling pathways and genes involved in breast muscle development, thereby refining the molecular mechanisms underlying skeletal muscle development in poultry.

This study revealed 42 genes whose expression differed across all growth stages, with some genes confirmed to be associated with muscle development. The *MYOM2* gene encodes myomesin-2, an important component of the sarcomeric M-band, and is highly expressed in chicken breast muscle and myocardium [36,37]. Myomesin-2 can bind to titin and myosin in the thick filaments, thereby enhancing the connections among thick filaments in skeletal muscle fibers and maintaining sarcomere stability [38]. In this study, the high expression of *MYOM2* on D1 may have sustained the high demand for myotome assembly driven by the increase in the number of myofibers during the embryonic period. The rebound in expression levels between D105 and D140 may be due to the accelerated rate of myofiber hypertrophy during the rapid growth phase, which requires reinforcement of the M-band structure and enhanced myofiber stability. Among the three breeds, *MYOM2* expression was downregulated only during the early developmental stage of XJ chickens, potentially reflecting breed-specific differences in muscle development requirements. *TM4SF1* promotes cell migration and growth while mediating cellular signaling, and knocking down *TM4SF1* inhibits proliferation and promotes differentiation in bovine myoblasts and C2C12 cells [39,40]. Furthermore, *Cyclin D1* is rapidly upregulated when muscle satellite cells enter the cell cycle, and studies have shown that overexpression of *TM4SF1* increases the expression level of *Cyclin D1* [41]. Therefore, *TM4SF1* may participate in the regulation of satellite cell proliferation by modulating *Cyclin D1* expression. The expression of *TM4SF1* in chickens was highest at D1 but then decreased, followed by a transient increase at D70 before declining again. This pattern suggests that a subset of satellite cells may remain proliferative initially. The brief rebound of *TM4SF1* during the middle stage may reflect its signaling function in response to muscle fiber hypertrophy during rapid weight gain. Furthermore, the expression levels of ENSGALG00010003777 and CCL21 increased continuously with age, suggesting that these genes may play crucial roles in muscle development, although their specific molecular mechanisms remain to be elucidated.

Muscle growth is a complex process influenced by multiple signaling pathways. KEGG analysis of comparative groups across different developmental stages revealed several pathways associated with growth, including focal adhesion, the regulation of the actin cytoskeleton, and the Wnt signaling pathway. Among these genes, those related to focal adhesion and the regulation of the actin cytoskeleton were enriched across all developmental stages. Focal adhesion involves complex protein assembly that mediates communication between the ECM and the intracellular actin cytoskeleton [42,43,44,45]. Focal adhesion plays an important role in myoblast differentiation, myofiber formation, and muscle hypertrophy through the PI3K/Akt signaling pathway [46,47,48]. IGF1R, a component of the focal adhesion pathway, is involved in PI3K/Akt signaling to promote muscle development [14,49]. In this study, *IGF1R* showed high expression at D1 and D140, suggesting that it may promote muscle development by activating PI3K/Akt signaling during the embryonic myofiber formation stage and the later stage of growth and development. The Wnt signaling pathway regulates skeletal muscle formation and regeneration, with the noncanonical Wnt signaling pathway playing a crucial role in promoting myofibrillar hypertrophy [50,51]. As a member of the Wnt family, *Wnt5a* effectively promotes myofibrillar hypertrophy and myotube formation via the noncanonical Wnt signaling pathway [52]. Overexpression of *Wnt5a* during the early stage of myofiber formation in chicks increases the number of slow-twitch muscle fibers [53]. In this study, *Wnt5a* expression was upregulated in XJ chickens, which may be one of the molecular bases underlying their superior meat quality. Additionally, mTOR signaling can positively regulate muscle hypertrophy and promote protein synthesis in skeletal muscle [54,55,56,57]. In this pathway, AKT3 promotes the proliferation and differentiation of chicken skeletal muscle satellite cells and enhances muscle protein accumulation and development in chickens via the L-Arg/NO/mTOR/p70S6K signaling pathway [58,59].

Through association analysis of genes and phenotypes, we can further identify key genes, thereby providing a crucial starting point for elucidating the molecular mechanisms underlying complex traits [60,61]. Ultimately, we identified the *NR4A3*, *HNMT*, and *MEGF10* genes. *NR4A3* promotes skeletal muscle hypertrophy and is involved in skeletal muscle oxidative metabolism [62,63]. *NR4A3* is significantly upregulated during the early developmental stages of multiple muscle tissues, suggesting that *NR4A3* may play a crucial role in the early hypertrophic processes of muscle cells. HNMT, a histamine N-methyltransferase, is widely distributed in the central nervous system [64]. Gu et al. reported that during late embryonic development in chicks, *HNMT* expression levels were significantly higher in broiler breast muscle than in layer breast muscle, indicating that the early establishment of muscle fiber morphology is accompanied by close interactions with the nervous system [65]. This study demonstrated that *HNMT* expression reached its highest level at D1 in all three chicken breeds, after which it declined progressively. This expression pattern may align with the active establishment of neural connections during the muscle fiber formation stage of embryonic development. The sustained decrease in expression levels after D1 may reflect the transition of neuromuscular junctions from active construction during the embryonic period to functional maturation after hatching. Additionally, *HNMT* can influence the content of flavor compounds such as anserine and carnosine in chicken meat, thereby helping to improve meat quality [66]. *MEGF10* is expressed in developing myoblasts and muscle satellite cells, and loss-of-function mutations in this gene cause recessive congenital myopathies [67,68,69]. *MEGF10* functions in the early stages of the satellite cell myogenic pathway, not only influencing myoblast proliferation and migration but also being significantly correlated with intramuscular fat content in chickens [70,71]. Similarly, *MEGF10* expression decreased with age across the three chicken breeds. The consistent expression patterns of the *HNMT* and *MEGF10* genes across multiple breeds indicate their universal functional roles in poultry muscle development. In summary, this study primarily analyzed the changes in gene expression levels in chicken breast muscle across different developmental stages. Future studies may consider integrating approaches such as metabolomics and single-cell RNA sequencing to more comprehensively elucidate the functional mechanisms, cellular heterogeneity, and regulatory networks of key genes [72,73].

## 5. Conclusions

In this study, genes such as *MEGF10*, *MYOM2*, *TM4SF1*, *HNMT*, *NR4A3*, and *Wnt5a* were identified, which may be involved in muscle development-related pathways, including the Wnt signaling pathway, focal adhesion, and the regulation of the actin cytoskeleton. Among these genes, *MYOM2* exhibited distinct expression trends during early XJ development, suggesting that this gene may be a key factor distinguishing XJ breast muscle development from that of other breeds. Conversely, the expression levels of *HNMT* and *MEGF10* gradually decreased with increasing age across all three chicken breeds, indicating that their functions may be universal in poultry muscle development. These findings provide a molecular basis for understanding the regulatory networks underlying breast muscle growth and offer candidate genes for molecular breeding. However, further functional validation is needed to elucidate the precise mechanisms by which these genes regulate muscle development.

## Data Availability

The mRNA sequenced reads generated in this study have been submitted to the NCBI SRA database as BioProject PRJNA1437386.

## Supplementary Materials

The following supporting information can be downloaded at https://www.mdpi.com/article/10.3390/ani16071071/s1. Figure S1: Gene expression analysis in high and low breast muscle weight samples at 140 days of age; Figure S2. Distribution of samples for breast muscle in CB and BJY chickens at 1 day of age; Figure S3. Distribution of samples for breast muscle in CB and BJY chickens at 91 days of age; Table S1: RT-PCR primer; Table S2: Phenotypic data of body weight (BW), body muscle weight (BMW), and breast muscle percentage (BMP) at five developmental stages; Table S3: Statistical analysis table of phenotypic data; Table S4: DEGs from inter-stage comparative groups; Table S5: Partial enriched pathway of C1, C2, C3, C4; Table S6: Genes of |KME| > 0.8 in different modules; Table S7: The enriched pathways of genes with |KME| > 0.8 in the greenyellow, darkgreen, darkolivegreen, and organe modules; Table S8: Correlation analysis between candidate genes and body weight, breast muscle weight, and breast muscle percentage.
