# Supplementary figures and images for: Comparative Analysis of the Transcriptome of the Chicken Breast Muscle at Different Developmental Stages
Source: Animals (Basel). 2026 Apr 1;16(7):1071. doi: 10.3390/ani16071071 (PMC13072039; doi:10.3390/ani16071071)

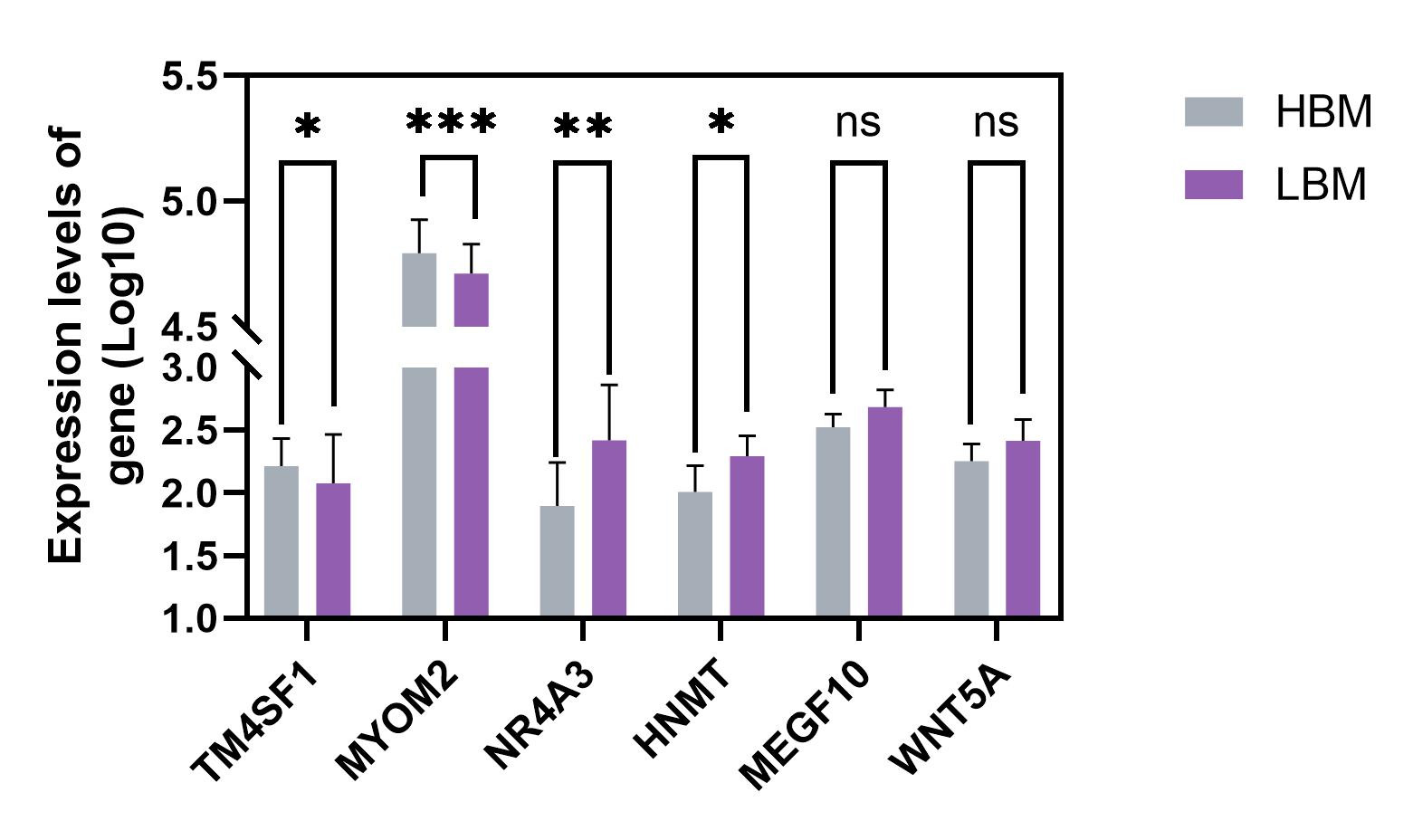

Supplement: Supplementary file 1 [file animals-16-01071-s001.zip › Figure S1.jpg]

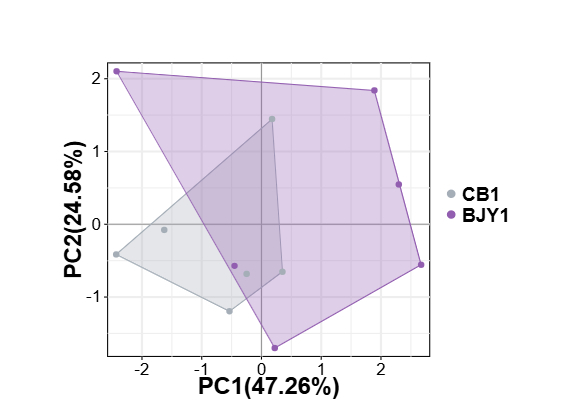

Supplement: Supplementary file 1 [file animals-16-01071-s001.zip › Figure S2.jpeg]

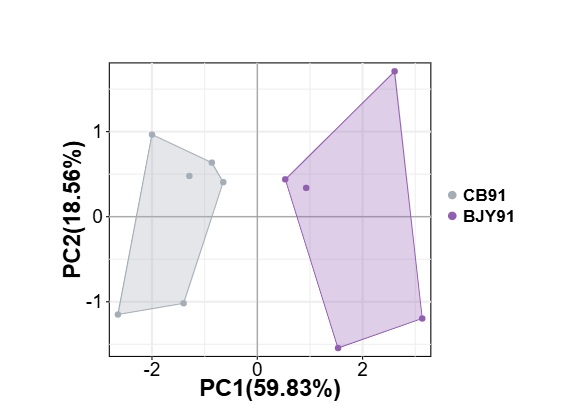

Supplement: Supplementary file 1 [file animals-16-01071-s001.zip › Figure S3.jpeg]
